# Can the level of HbA_1_C predict diabetic retinopathy among type II diabetic patients?

**DOI:** 10.1186/s12886-022-02608-3

**Published:** 2022-10-31

**Authors:** Javad Setareh, Ghazale Hoseinzade, Batoul Khoundabi, Mahsa Kamali, Ata Ebrahimi, Andarz Fazlollahpour-Naghibi, Mohammad Zareei, Mona Mohamaditabar, Ali Makaremi

**Affiliations:** 1 Psychiatry and Behavioral Sciences Research Center, Addiction Institute, Mazandaran University of Medical Sciences, Sari, Iran; 2 Faculty of Medicine, Sari branch, Islamic Azad University, Sari, Iran; 3 Iran Helal Institute of Applied-Science and Technology, Tehran, Iran; 4 Research Center for Health Management in Mass Gathering, Red Crescent Society of the Islamic Republic of Iran, Tehran, Iran; 5 Pediatric Infectious Diseases Research Center, Communicable Diseases Institute, Mazandaran University of Medical Sciences, Sari, Iran; 6 Department of ophthalmology, Faculty of medicine, Sari branch, Islamic Azad University, Sari, Iran

**Keywords:** Diabetes, Retinopathy, HbA_1_C, Epidemiology, Diagnosis

## Abstract

**Background:**

Hemoglobin A1C (HbA_1_C) test is the best care evaluation measurement due to a strong correlation between the test results and diabetic complications. So, this cross-sectional study aimed to assess whether the level of HbA_1_C can predict Diabetic Retinopathy (DR) among Type 2 diabetes mellitus (T2DM) in the Iranian population.

**Method:**

One hundred sixty-eight diabetic patients were selected via the convenience sampling method. Data were collected by research made questionnaire scale and laboratory test had been done. To estimate the cut off point for some variables statistical tests, formal measures of classification performance, model evaluation criteria and a decision Tree were used.

**Results:**

The prevalence of DR was 29.8%. The Receiver Operating Characteristic (ROC) curve and decision tree showed the optimal cut-off point for the HbA1C variable that separates the patient with and without DR is HbA_1_C = 8.15.

**Conclusion:**

Current study showed an appropriate cutoff point for detecting the development of DR among diabetic patients. So, this cutoff point can be used as guide evidence in several clinical judgments on the Iranian population.

## Background

Type 2 Diabetes Mellitus (T2DM) is defined as relative insulin deficiency secondary to pancreatic β-cell dysfunction and also insulin resistance in specific organs [1]. The overall increasing trend has been predicted in diabetic prevalence so that will increase from 415 million patients in 2015 to 642 million patients in 2040 all over the world [2]. Another report showed the prevalence of diabetic patients increased by 69% in developing countries from 2010 to 2030 [3]. Based on a World Health Organization (WHO) report in 2016, the prevalence of type 2 diabetes in Iran was 10.3% [4] and the Iranian population pays vast sums for the treatment of diabetes-related health complications [1]. The complications of diabetes mellitus are divided to macro vascular complications such as cardiovascular disease with 50% prevalence and micro vascular complications related to the kidney, the retina and the nervous system, which involved 27% of T2DM patients [5]. Diabetic Retinopathy (DR) is one of the major micro vascular complications of the diabetic disease and also divided into two stages, including Non-Proliferative Diabetic Retinopathy (NPDR) and Proliferative Diabetic Retinopathy (PDR) that occur in the early stage and advanced stage of DR, respectively [6]. Diabetic Macular Edema (DME) has been recognized as swelling and thickening of the macula and is the most prevalent cause of blindness among DR patients [7]. A retrospective cohort study revealed eyes with moderate NPDR, severe NPDR, and PDR were more likely to develop sustained blindness after 2 years of DR diagnosis versus others [8]. Hence assessing these complications is important. The development of DR is a multiplex and multidimensional process, so the diagnosis of DR is challenging [9, 10]. A systematic review in Iran showed the prevalence of DR, NPDR and PDR were 41.9, 32.2 and 13.2%, respectively [11]. Another systematic review revealed NPDR is more common among Asian DR patients so screening management is crucial [12]. Nevertheless, 45.8% of all diabetic patients were undiagnosed and so untreated. It causes diabetic patients at higher risk of developing complications [13, 14]. Hence, early detection of T2DM is the main factor for optimal outcomes by preventing or delaying the development of complications [15]. World Health Organization (WHO) reported some cost effective interventions that can enhance diabetic patient outcomes such as regular screening for damage to the eyes [16]. Glycated hemoglobin (HbA1c) reflects the chronic blood glucose concentration and it is used as an index to reflect the average blood glucose levels of the past 1–2 months [17]. HbA1c test is the best care evaluation measurement due to a strong correlation between the test results and the diabetic complications [18]. A study revealed when HbA1c was≥6.8%, the odds ratio for diabetic retinopathy increased significantly [17]. Another study showed the cut off 6.6% is the best detected for the presence of any DR [19]. Sumner et al. believed that various risk factors could affect the results of HbA1c [20].

Given the importance of screening DR among T2DM to reduce complications and enhance the quality of life, using the diagnostic criteria may be essential to help health care providers. Also, the relationship between HbA_1_C and DR is race dependent [17]. However, among the Iranian population this cutoff point is unclear. So, the purpose of this cross-sectional study was to assess whether the level of HbA_1_C can predict DR among T2DM in the Iranian population.

## Methods

### Study design and setting

This cross-sectional study was conducted on Type 2 diabetes mellitus (T2DM) patients where referred to the outpatient clinic between March 2019 and February 2020 in Mazandaran province, Iran. The outpatient clinic is affiliated to one of the teaching hospitals in the north of Iran, and people could refer to this clinic for treatment. Among these people, those were under the DM treatment selected.

### Sample and sampling method

**Fig. 1:**
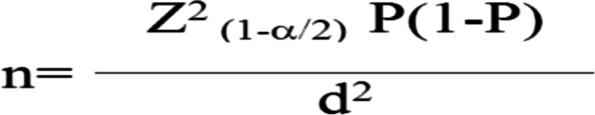
Sample size formula

Participants were selected via the convenience sampling method, and the sample size was calculated 147 patients following the results obtained by Yao et al. study [21], considering α = 0.05 and error equal to 0.07 (Fig. 1). Patients who had informed consent to participate, aged at least 18 years old, known treated diabetes disease at least 1 year and had the ability to communicate were included. Type I diabetes patients, pregnant women and subjects missing Fasting Plasma Glucose (FPG), HbA1c or fundus photography were excluded. In this study Diabetes was defined as self-reported of a previous diagnosis of the disease, use of diabetic medications, or HbA1c 6.5% or greater.

### Measurements instrument

Data were collected by three parts questionnaire. The first part was a researcher-made socio-demographic questionnaire including family history, medical history, age, gender, etc. One of the researchers completed this part via face-to-face interviews. The second part included physical examination such as measurement of height, weight and waist circumference performed by one of the researchers. In this part also, laboratory tests consisted of FPG, HbA1c, Cholesterol (Chol), Triglyceride (TG), High-Density Lipoprotein (HDL), Low-Density Lipoprotein (LDL), Blood Urea Nitrogen (BUN), Creatinine [1], etc. had been done after a minimum overnight fasting of 12 hours. FPG and HbA1c were determined by the chromatography method.

### Data collection

After completing these two-section, one of the researchers made a telephone call to all the T2DM patients to make an appointment for a clinical visit in the ophthalmology clinic in the same hospital. On the second visit, the third part of the questionnaire was completed. During this visit, Best Corrected Visual Acuity (BCVA), by slit lamp bio microscopy and Optical Coherence Tomography (OCT) were performed for both eyes by a specialist. Also, Fundus photographs (FP) were taken to evaluate of the presence and signs of DR and Diabetes Macular Edema (DME).

In the ophthalmology visits after pupil dilatation with 1.0% tropicamide and 10% phenylephrine, fundus photographs (45° color digital images of the retina) were taken from both eyes of each participant by a technologist using a Topcon TRC-NW7SF fundus camera (Topcon corporation, Tokyo, Japan). The first image was centered on the macula, whereas the second one was centered on the optic nerve. The photographs were assessed according to the international clinical DR severity scale [22] by photographic graders blinded to the clinical information of the participants. In the present study the kappa coefficient between two graders was 0.89. This scale rates DR at five different levels: (i) no retinopathic changes (Equivalent to the early Treatment of Diabetic Retinopathy Study (ETDRS); scale level 10); (ii) mild Non-Proliferative Diabetes Retinopathy (NPDR); equivalent to ETDRS level 20; (iii) moderate NPDR (equivalent to ETDRS levels 35, 43, and 47); (iv) severe NPDR (equivalent to ETDRS levels 53A-53E); and (v) Proliferative Diabetes Retinopathy (PDR) (equivalent to ETDRS levels 61 or higher). As mentioned previously, the presence of DR was defined as the presence of moderate (level iii) or severe non-proliferative (level iv) DR, or proliferative (level v) DR in each eye.

### Data analysis

Finally, the patients were divided into two groups (with DR and without DR). Then a comparison between them was made in terms of variables. The data were analyzed using the statistical package IBM SPSS version 24.0 (Statistical Package for the Social Sciences, Chicago, IL). Kolmogorov-Smirnov test was applied to test the normality distribution for quantitative variables. To explore the independent nature of some categorical variables, chi-square or exact Fisher tests were used. The comparison of the means between two groups was done by independent t-test for age and Mann-Whitney test for the rest of the variables.

Formal measures of classification performance and model evaluation criteria including sensitivity, specificity and positive and negative predictive values were considered. The Area Under the Curve (AUC) of a Receiver Operating Characteristic (ROC) curve is a way to reduce the ROC performance to a single value representing the expected performance. Decision Tree as a nonparametric method, was used to estimate the cut off points for some studied variables. It uses the Classification and Regression Tree according to the specific variables used (Continues and Categorical) by Classification and Regression Tree (CART) and Chi-squared Automatic Interaction Detection (CHAID).

Statistical significance mainly depends on the sample size, the data’s quality and statistical procedures’ power. Then, we used the effect sizes to describe the strength of a phenomenon. The most popular effect size measure surely is Cohen’s d (Cohen, 1988) used in this research.

## Results

### Socio-demographic characteristics of T2DM patients

Of a total of 327 diabetes patients, 168 participants were assessed and received eyes examination. The results revealed most of the patients were female (68.5%) and mean ± SD age and duration of DM were 56.01 ± 10.79 and 11.61 ± 7.58 years, respectively. The prevalence of DR was 29.8%. Most of the participants had moderate NPDR with the prevalence of 40.0 and 36.0% in OS and OD, respectively (Table 1).

**Table 1:** Socio-demographic characteristics of T2DM patients

Variables	Patients(*n* = 168)	
Gender	Male	53 (31.5%)
	Female	115 (68.5%)
DR	Yes	50 (29.8%)
	No	118 (70.2%)
Severity of DR (OS)	Mild NPDR	11 (22.0%)
	Moderate NPDR	20 (40.0%)
	Severe NPDR	12 (24.0%)
	PDR	7 (14.0%)
Severity of DR (OD)	Mild NPDR	13 (26.0%)
	Moderate NPDR	18 (36.0%)
	Severe NPDR	12 (24.0%)
	PDR	7 (14.0%)
Age (year)	56.01 ± 10.79	
Duration of DM (year)	11.61 ± 7.58	

Categorical and quantitative items have been shown by n (%) and Mean ± SD respectively

*DR* Diabetic Retinopathy, *OD* Oculus Dexter *OS* Oculus Sinister, *SD* Standard Deviation, *DM* Diabetes Mellitus, *NPDR* Non-Proliferative Diabetic Retinopathy, *PDR* Proliferative Diabetic Retinopathy

### Comparison of demographic variables and complications stratified by DR and severity of DR

Patients with and without DR differed significantly in terms of age, Years of Affiliations (YOA), HbA_1_C, Visual Acuity Right (VAR), Visual Acuity Left (VAL), thyroid disease and the history of cataract and pterygium (*P* < 0.05; Table 2). But the severity of DR differed significantly in terms of HbA_1_C, history of GI disease and pterygium (*P* < 0.05; Table 3).

**Table 2:** Comparison demographic variables, micro-vascular, macro-vascular complications and refractive errors among patients with and without DR

Variable	Group		*p*-value
	With DR *n* = 50	Without DR *n* = 118	
Gender	12(24.0)	41(37.4)	0.205*
Insulin	10(20.8)	13(11.0)	0.082*
Age, (Year)	59.6 ± 6.3	54.5 ± 11.9	0.002**
BMI (Kg/m^2^)	28.2 ± 6.1	29.5 ± 4.0	0.108**
YOA	14.0 ± 7.8	10.7 ± 7.4	0.006***
HbA_1_C	8.5 ± 1.8	7.8 ± 1.6	0.007***
FBS	166.0 ± 56.8	165.1 ± 61.4	0.718***
BS	250.0 ± 97.3	231.1 ± 100.3	0.311***
BUN	23.4 ± 10.7	20.6 ± 10.3	0.056***
Cr	1.1 ± 0.4	1.6 ± 6.5	0.064***
HDL	44.7 ± 11.4	44.0 ± 12.8	0.498***
LDL	84.6 ± 26.4	88.1 ± 40.2	0.936***
TG	170.5 ± 90.3	183.8 ± 115.3	0.661***
Chol	165.4 ± 67.2	172.7 ± 59.8	0.369***
VAR	6.2 ± 2.9	7.2 ± 2.8	0.024***
VAL	6.0 ± 2.9	7.3 ± 2.6	0.002***
HTN	29(58.0)	65(55.1)	0.738*
Thyroid Disease			
Hypothyroidism	13(26.0)	12(10.2)	0.022*
Normal	37(74.0)	103(87.3)	
Hyperthyroidism	0(0.0)	3(2.5)	
GI	30(60.0)	53(44.9)	0.092*
ME	11(22.0)	4(3.4)	< 0.001*
PR	7(14.0)	12(10.2)	0.474*
Cat	27(54.0)	41(34.7)	0.025*
P	14(28.0)	5(4.2)	< 0.001*
IOP L	4.3 ± 3.6	4.8 ± 3.7	0.671***
IOP R	4.2 ± 3.6	4.8 ± 3.6	0.642***

Categorical and quantitative items have been shown by n (%) and Mean ± SD respectively

*BMI* Body Mass Index, *HbA*_*1*_*C* Hemoglobin A1C, *FBS* Fasting Blood Sugar, *BS* Blood Sugar, *BUN* Blood Urea Nitrogen, *Cr* Creatinine, *HDL* High-Density Lipoprotein, *LDL* Low-Density Lipoprotein,*TG* Triglyceride, *Chol* Cholesterol, *IOP* Intra Ocular Pressure, *R* Right, *L* Left, *Cat* Cataract, *ME* Macular Edema, *HTN* Hypertension, *YOA* Years of Affiliations, VAR Visual Acuity Right, *VAL* Visual Acuity Left, *GI* Gastrointestinal, *P* Pterygium, *SD* Standard Deviation

*Chi-square **T-test ***Mann-whitney

**Table 3:** Comparison of demographic variables, micro-vascular and macro-vascular complications in patients with DR stratified by severity of disease

	Group				
Items	Mild NPDR *n* = 14	Moderate NPDR *n* = 18	Severe NPDR *n* = 12	PDR *n* = 6	*p*-value
Gender	5(35.7)	5(27.8)	1(8.3)	1(16.7)	0.328*
Insulin	2(14.2)	4(22.2)	2(16.7)	2(33.3)	0.882*
Age	60.7 ± 6.6	59.9 ± 7.0	59.0 ± 6.0	59.5 ± 4.1	0.899**
BMI (Kg/m^2^)	27.7 ± 8.7	28.8 ± 5.0	29.2 ± 3.9	26.8 ± 4.8	0.827**
YOA	15.4 ± 8.0	12.4 ± 6.7	15.8 ± 8.2	13.3 ± 10.5	0.498***
HbA_1_C	8.9 ± 1.3	8.0 ± 1.2	9.2 ± 1.4	7.8 ± 1.6	0.029*
FBS	165.2 ± 75.1	154.6 ± 41.9	183.2 ± 59.7	151.8 ± 54.6	0.583***
BS	296.3 ± 117.7	218.7 ± 78.1	247.7 ± 90.2	240.5 ± 108.5	0.204***
BUN	26.0 ± 14.0	23.4 ± 9.7	21.8 ± 9.6	22.7 ± 11.0	0.894***
Cr	1.0 ± 0.2	1.1 ± 0.4	1.0 ± 0.3	1.2 ± 0.4	0.621***
HDL	46.6 ± 13.6	43.4 ± 10.0	42.7 ± 13.4	45.0 ± 6.3	0.912***
LDL	79.1 ± 27.1	84.0 ± 21.5	75.9 ± 26.8	94.5 ± 11.6	0.206***
TG	168.3 ± 60.0	186.7 ± 92.4	177.9 ± 126.7	125.7 ± 55.0	0.450***
Chol	161.8 ± 105.0	160.9 ± 31.6	165.0 ± 79.3	164.2 ± 26.0	0.721***
VAR	6.8 ± 3.4	6.8 ± 2.3	6.4 ± 2.5	3.2 ± 3.1	0.133***
VAL	6.3 ± 3.3	6.4 ± 2.6	6.3 ± 2.7	3.7 ± 2.9	0.285***
HTN	9(64.3)	11(61.1)	6(50.0)	2(33.3)	0.364*
Tyr (Hypothyroidism)	3(21.4)	4(22.2)	4(33.3)	1(16.7)	0.932*
GI	9(64.3)	6(33.3)	9(75.0)	5(83.3)	0.026*
ME	2(14.2)	3(16.7)	3(25.0)	2(33.3)	0.817*
PR	2(14.2)	3(16.7)	2(16.7)	0(0.0)	0.840*
Cat	7(50.0)	11(61.1)	6(50.0)	3(50.0)	0.975*
P	1(7.1)	8(44.4)	4(33.3)	0(0.0)	0.047*

Categorical and quantitative items have been shown by n (%) and Mean ± SD respectively SD: Standard Deviation

*Chi-square **ANOVA ***Kruskal–Wallis

### Optimal cut-off point for the HbA_1_C variable in T2DM

**Fig. 2:**
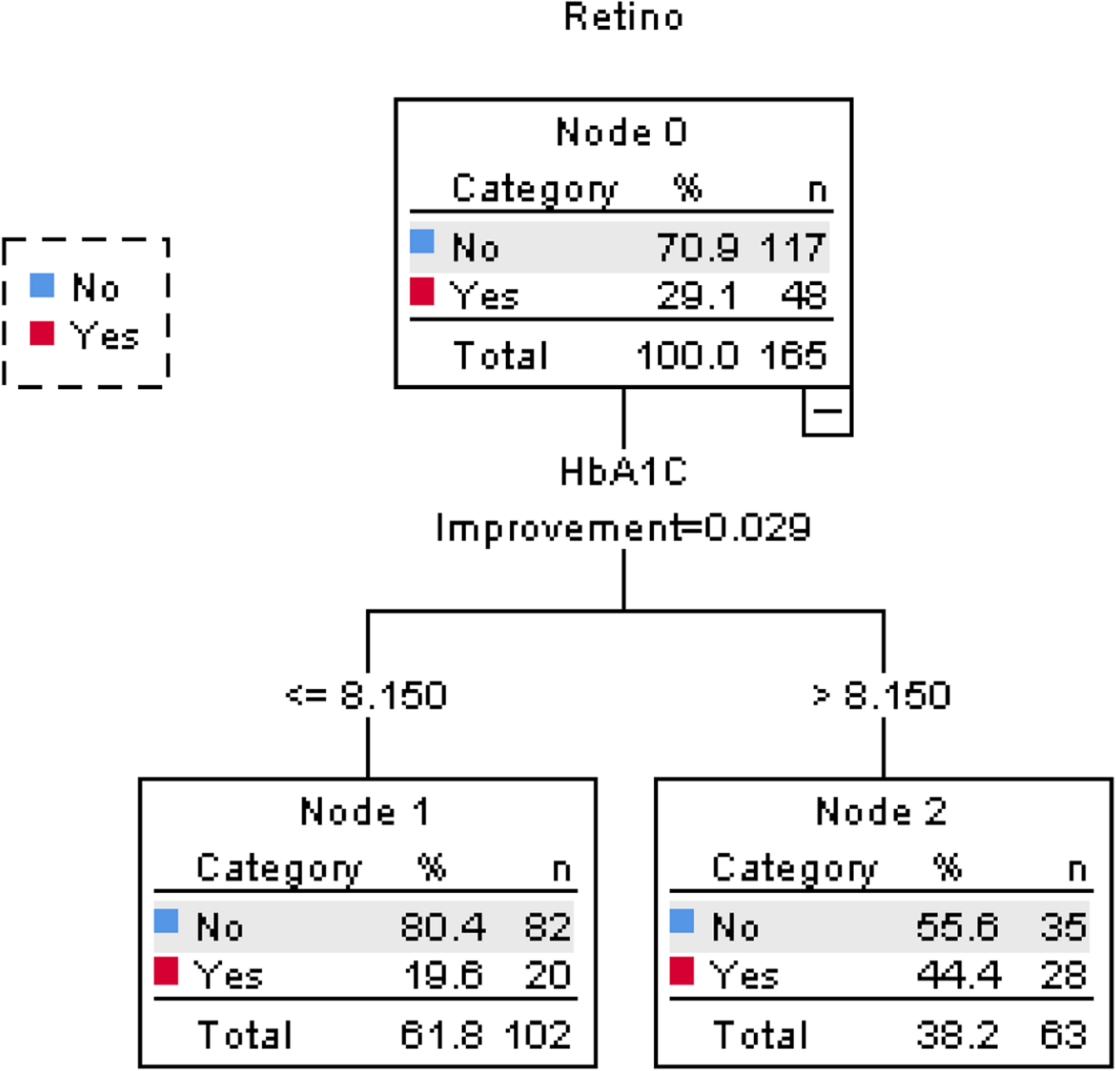
Decision tree classification of patients with and without DR based on HbA_1_C

**Fig. 3:**
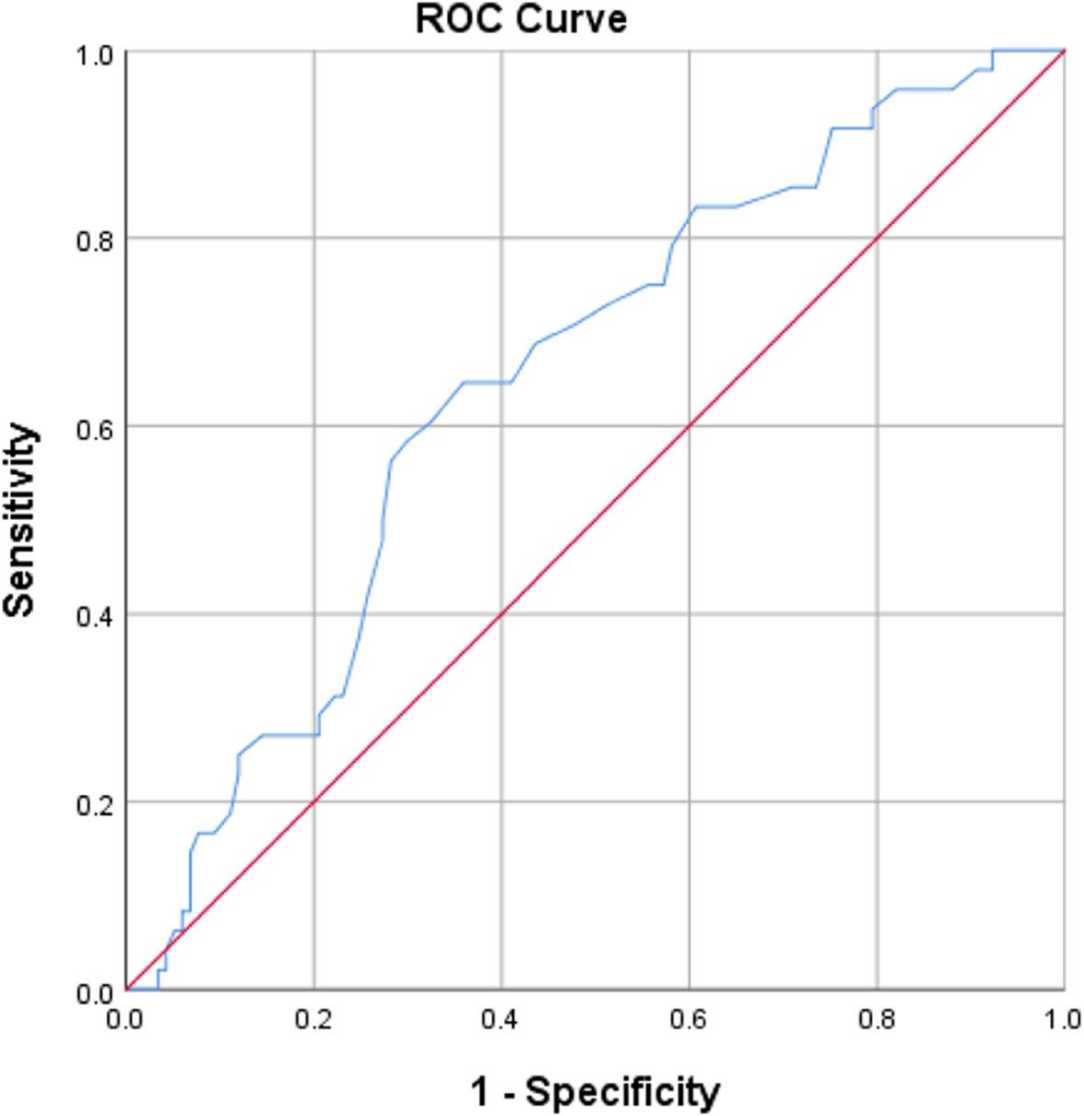
ROC curve for HBA1c values as screening test. DR: Diabetic Retinopathy

ROC curve and decision tree (Figs 2 and 3) showed the optimal cut-off point for the HbA1C variable that separates the patient with and without DR is HbA_1_C = 8.15, which leads in a sensitivity and specificity of 0.583 and 0.701, respectively (Table 4 and Fig 2). This high-specific cut-off point well identifies patients without retinopathy in the first stage of disease.

**Table 4:** Cut-off point for HbA1C variable

Positive if Greater Than or Equal To	Sensitivity	1 - Specificity
5.45	1.000	0.957
5.95	0.979	0.923
6.45	0.958	0.821
6.95	0.854	0.709
7.35	0.750	0.573
7.85	0.646	0.410
8.05	0.604	0.325
8.15	0.583	0.299
8.35	0.500	0.274
8.85	0.313	0.231
9.25	0.271	0.171
9.95	0.167	0.094
10.75	0.063	0.060
11.85	0.000	0.034
12.25	0.000	0.017

## Discussion

The purpose of the present study was to determine whether the level of HbA_1_C can predict DR among T2DM. In our study, the prevalence of DR among T2DM patients was 29.8%. This was nearly similar to the study conducted in one of the north provinces in Iran. In this study, the prevalence of DR among diabetic patients was 23.8% [23]. In Iran’s other meta-analysis the overall prevalence of DR among T2DM was 37.8% [3]. Also, the prevalence of DR was 33.5% in the Chinese population [24]. While, it was 18.58% in another study in china [25]. However, the prevalence of DR was higher among Saudi Arabia’s population. This study revealed the prevalence of DR was 40.0% [26].

In this cross-sectional study, ROC curve analysis and decision tree showed the appropriate cutoff point for detecting DR was HbA_1_C equal to 8.15. This value is similar to Aziz et al. study. He explored the cutoff point of 8.9% for developing DR with 67% sensitivity and 50% specificity. But this value was lower in other studies [26]. A study demonstrated that the optimal HbA_1_C threshold for detecting DR was 6.6% [19]. Iranian population-based study showed that optimal cut-off point of HbA_1_C was 6.2% [27]. In the previous study, one of the reasons for limited comparison was the discrepancy in participants. They assessed all diabetic and non- diabetic patients and people at least 40 years old. Based on the results of other studies, diversity in the optimal level of HbA_1_C for detecting DR might be due to taking an anti-hyperglycemic medication. An Egyptian study demonstrated that cut-off point level of HbA_1_C is higher among diabetic patients than non-diabetic people. It was 6.9% of the population, but after excluding consumers of anti-hyperglycemic medication the value changed to 7.5% [28]. This finding also had been seen in Xin et al. study. They showed the appropriate cut off point in the general population was 6.4%, but after modifying anti-hyperglycemic medication, consumers this value reached 6.7% [29]. Other reason that justify these difference is the possible effects of inadequate vitamin D. Long et al. reported that insufficient vitamin D might increase the risk of severe DR even in patients with well- controlled glycemia [30]. This possibility was missed in mentioned studies.

The limitation of the present study was the use of the population of one region, which limited the generalization of finding. Therefore, we suggest assessing more variables that may affect the relationship between DR and the level of HbA1c for future studies in this regard. One of the important risk factors is anemia. The literature review showed anemia may lead to diabetic retinopathy without renal disease [31, 32]. So, it is recommended to assess this variable in the future studies.

## Conclusion

This cross-sectional study showed the prevalence of DR was 29.8% among T2DM patients. Also, the appropriate cut off point of HbA_1_C for detecting DR as a disabling complication is 8.15 among Iranian diabetic population. However, additional studies that modify confounding variables are needed to confirm the appropriate level of HbA_1_C for detecting the development of DR among diabetic patients.

## Data Availability

The datasets used and/or analyzed during the present study are available from the corresponding author on reasonable request.
